# Review of Ecologically-Based Pest Management in California Vineyards

**DOI:** 10.3390/insects8040108

**Published:** 2017-10-11

**Authors:** Houston Wilson, Kent M. Daane

**Affiliations:** 1 Department of Entomology, University of California, Riverside, Riverside, CA 92521, USA; 2 Department Environmental Science, Policy and Management, University of California, Berkeley, Berkeley, CA 94720-3114, USA; kdaane@ucanr.edu

**Keywords:** grapes, vineyard, integrated pest management, mating disruption, habitat management, natural enemy augmentation, animal integration, conservation biological control, biodynamic preparations

## Abstract

Grape growers in California utilize a variety of biological, cultural, and chemical approaches for the management of insect and mite pests in vineyards. This combination of strategies falls within the integrated pest management (IPM) framework, which is considered to be the dominant pest management paradigm in vineyards. While the adoption of IPM has led to notable and significant reductions in the environmental impacts of grape production, some growers are becoming interested in the use of an explicitly non-pesticide approach to pest management that is broadly referred to as ecologically-based pest management (EBPM). Essentially a subset of IPM strategies, EBPM places strong emphasis on practices such as habitat management, natural enemy augmentation and conservation, and animal integration. Here, we summarize the range and known efficacy of EBPM practices utilized in California vineyards, followed by a discussion of research needs and future policy directions. EBPM should in no way be seen in opposition, or as an alternative to the IPM framework. Rather, the further development of more reliable EBPM practices could contribute to the robustness of IPM strategies available to grape growers.

## 1. Introduction

There are 560,000 bearing acres of wine grapes in California with an annual farm-gate value of approximately $2.5 billion dollars, which makes it one of the state’s top five commodities [1]. California wine grape growers employ a variety of sophisticated pest control practices within an integrated pest management (IPM) framework. Improved decision-making and timing of chemical controls, along with the introduction of novel pesticide chemistries with more selective modes of action, paired with improvements to spray application technology, have all reduced the environmental impact of wine grape production. That said, more than 26 million pounds of active ingredients are still applied to this crop annually. Sulfur is the leading chemical applied, followed by other fungicides, herbicides, and insecticides. For insecticides, the top active ingredients used are imidacloprid, abamectin, methoxyfenozide and spirotetramat [2].

A combination of regulatory pressure [3], consumer demand, and personal interest continues to motivate growers to reduce the human and environmental impacts of wine grape production in California. While IPM remains the dominant paradigm, many growers have been experimenting with a subset of IPM practices referred to as ecologically-based pest management (EBPM), which makes use of mating disruption, habitat manipulation, natural enemy augmentation and conservation, animal integration, and other non-pesticide alternatives. While these practices were inherent in the original descriptions of IPM, many have argued that they have been lost or ignored in the translation of IPM theory to IPM practice [4,5,6]. Subsequently, the term EBPM has been used to distinguish these practices from the broader term IPM, although it is important to note that EBPM and IPM are not mutually exclusive. This most notably resulted in the further definition and outline of EBPM strategies by the National Research Council in the mid-1990s [7]. While promising, many of these strategies are woefully understudied and/or unsupported by current agricultural policy. 

What follows is a broad overview of IPM in California wine grape vineyards, and the key EBPM strategies being employed. These strategies have been grouped into the following categories: (1) pheromone mating disruption, (2) ant control for mealybugs, (3) habitat management, (4) natural enemy augmentation, (5) animal integration, and (6) biodynamic preparations. For both IPM and EBPM, general practices and efficacy are described, and an estimate is provided of how commonly these strategies are employed in vineyards (Table 1). In many cases, the literature on these EBPM practices is sparse, with little available experimental evidence. As such, the article concludes with an outline of future research needs and policy to support the further development and implementation of EBPM programs.

## 2. Insect and Mite Pest Management Practices

### 2.1. Integrated Pest Management in California Wine Grape Vineyards

Integrated pest management is an ecosystem-based strategy that focuses on the long-term prevention of crop pests and diseases through a variety of techniques that include biological control, the use of resistant varieties, habitat management, modification of cultural practices and, when needed, judicious and timely use of chemical controls [8,9,10]. On the ground, IPM functions as a decision-making framework to address immediate and localized pest infestations in the most economical manner possible. As a whole, this is a field data- and science-driven approach to pest and disease management that aims to reduce the need for, and impacts of, pesticide use in agriculture for both economic and environmental benefit. When chemical controls are deemed necessary, product selection, timing, and application methods are designed to maximize efficacy against the pest or disease, while minimizing impacts on natural enemies and other non-target organisms.

Almost all of the wine grape growers in California subscribe to some form of IPM. With the development and addition of IPM curricula into mainstream viticulture programs in the 1970s/80s, it has effectively become a de facto practice for the current generation of wine grape growers. For most operations, it is standard practice to design and develop new vineyards with an IPM approach in mind. For example, rootstock and cultivar selection can play an important role in determining the pest colonization of the vineyard. Growers can select rootstocks resistant to grape phylloxera (*Daktulosphaira vitifoliae*) and nematodes (*Meloidogyne* spp.) [11,12]. Cultivars also vary in their susceptibility to mites [13] and leafhoppers [14].

Once established, vineyards are regularly monitored for insect and disease pressure. These data are then used in conjunction with publicly available resources on best management practices (e.g., UC IPM http://ipm.ucanr.edu/) to make informed management decisions. In many cases, commercial operations may even include specialized employees (i.e., pest control advisors) to carry out monitoring and provide the head viticulturalist with management recommendations as well as oversee their implementation in the vineyard.

Research plays a key role in developing effective IPM strategies. Studies on the ecology and biology of pests and their natural enemies have improved pest management by identifying vulnerabilities in pest life cycles and then adjusting practices to maximize their impact on pests. For instance, vine mealybug (*Planococcus ficus*) populations are more exposed on the grape vine at certain times of the year [15]; this information can be used by growers to improve scouting and spray timing. In another example, early-season populations of *Erythroneura* leafhoppers are known to deposit their eggs into the available leaf tissue on the grape vine, some of which will be removed by growers to promote proper canopy development [16]. By timing these practices to follow peak *Erythroneura* leafhopper egg deposition, it may be possible for growers to reduce the number of viable leafhopper eggs in the vineyard. The removal of basal leaves in particular is common to reduce the incidence of powdery mildew [17], although more research is needed to document the impact of this practice on leafhoppers.

When chemical controls are needed, improved equipment such as airblast and electrostatic sprayers, or the application of systemic materials through the irrigation system, have allowed growers to improve coverage and reduce pesticide drift [18]. Furthermore, the introduction of “reduced risk” and/or “organophosphate or carbamate alternative” pesticides that make use of novel chemistries and modes of action (e.g., spirotetramat) can potentially reduce non-target impacts and/or the overall quantity of chemical controls applied in the vineyard. To be clear, these products are not entirely benign. For instance, many systemic pesticides are still known to have some impacts on non-target organisms [19,20,21], and current evaluation of novel pesticides may overlook sublethal effects and more complex impacts on arthropod communities [22,23,24,25]. Growers certified under the USDA National Organic Program (NOP) and/or Demeter^®^ biodynamic are limited to the use of materials that have been certified by the Organic Materials Research Institute (OMRI), which in wine grape production primarily consists of botanical pesticides such as pyrethrin, azadirachtin and spinosad [26]. These products can be effective, but are hampered by rapid degradation in the field and their broad insect toxicity, which leads to non-target impacts on natural enemies [27,28,29]. These factors can create the need for multiple follow-up applications and cause secondary pest outbreaks [30,31]. While IPM does not eliminate pesticide use, it has certainly reduced it, and contributed to the more effective and judicious use of chemical controls in modern viticulture.

### 2.2. Mating Disruption

The use of synthetic insect pheromones as part of a pest mating disruption program is common in many perennial cropping systems. In California wine grapes, pheromone traps are commonly used to monitor—and mating disruption is commonly used to control—populations of vine mealybug (*Planococcus ficus*) [32,33,34]. Similar approaches are available for some Lepidopteran pests, including the European grapevine berry moth (*Lobesia botrana*), omnivorous leafroller (*Platynota stultana*), and orange tortrix (*Argyrotaenia franciscana*) [35,36,37].

Pheromone mating disruption can be easily integrated into modern vineyards; as a result, wine grape growers are increasingly using it for the management of vine mealybug. This approach is not available for many other key pests, since they either do not rely as heavily on pheromones for reproduction (e.g., leafhoppers), their reproduction does not necessarily rely on mating (i.e., arrhenotokous pests), and/or populations are typically too high for mating disruption to be effective (e.g., mites). That said, some alternatives are in development. For instance, leafhopper mating is initiated through a series of vibrational signals sent over the leaf surface, and there is currently research in progress to develop modes of interrupting this communication process by transmitting disruptive vibrational signals to the grape vine through trellis wires using an electromagnetic shaker [38,39].

### 2.3. Ant Controls for Mealybugs

The biological control of mealybugs and aphids can be impeded by the presence of ants, which feed on pest excrement (i.e., “honeydew”) and in turn protect them from natural enemies. In wine grape systems, Argentine ants (*Linepithema humile*) in particular are known to protect a variety of mealybug species, including obscure (*Pseudococcus viburni*), grape (*Pseudococcus maritimus*), long-tailed (*Pseudococcus longispinus*), and vine mealybug (*Planococcus ficus*). The use of bait stations has been shown to be an effective means of reducing ants in the vineyard, which can lead to improved natural enemy impact on mealybugs [40,41,42,43].

While bait stations do not require any type of fundamental change in the structure of the vineyard itself, high densities of bait stations are needed for effective control in a vineyard, which can be an impediment for larger operations farming hundreds or thousands of vineyard hectares. Given the low cost and efficacy of chemical controls for mealybugs, most growers do not use ant bait stations, although there is interest and ongoing research in developing better modes of bait delivery [44].

### 2.4. Habitat Management

The use of monoculture cropping practices has been identified as an underlying contributor to pest and disease outbreaks in agriculture due to the concentration of plant host resources for pests, and the absence of a non-crop habitat necessary to support natural enemy populations [45,46]. The addition of non-crop plants, especially flowering species, can attract and maintain natural enemies in agricultural settings [47,48,49], and in some cases, this increase in agrobiodiversity can lead to increased ecosystem services, including the biological control of pests [50,51]. Polyculture systems comprised of multiple crops or crop varieties can act in a similar way [52].

Perennial hedgerows and annual cover crops are the most popular forms of habitat provision in vineyards. Most common is the use of overwintering cover crops, which are sown in vineyards each fall after harvest to provide soil cover during winter rains, and to improve soil quality. Most wine grape growers do this with some regularity. These cover crops usually consist of legume/grass blends (these include legumes such as *Vicia* spp., *Vigna* spp., and *Trifolium* spp.; and grasses such as *Secale cereale*, *Avena sativa*, and *Hordeum vulgare*). While these covers can attract and support a variety of natural enemies that are active early in the season [53], they are typically mown and incorporated into the soil in the spring right around bud break, which minimizes their utility for pest management during the grape growing season. One exception is the use of mustards (e.g., *Brassica* spp.) as an overwintering cover, which has been shown to have some residual impact on nematode populations as it breaks down in the soil following tillage [54].

Permanent ground covers, usually species of short grass (e.g., *Lolium perenne*, *Bromus hordeaceus*, *Vulpia myuros*), are periodically established in vineyards with high soil fertility to regulate vine vigor. While not primarily used as a pest management tool, lower vigor vines can be less attractive to certain pests, such as leafhoppers [53]. Perennial ground cover can also reduce dust, which can induce mite outbreaks [55,56,57].

**Figure 1:**
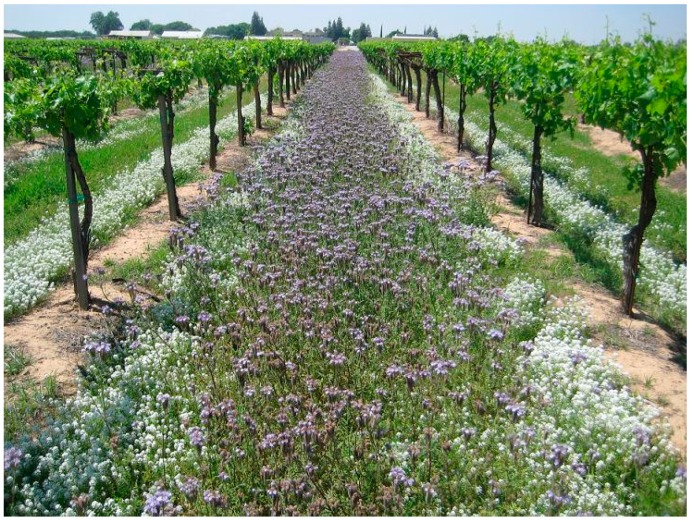
Summer flowering cover crops *Phacelia tanacetifolia* and *Lobularia maritima* in a vineyard.

Summer flowering cover crops have greater potential to support natural enemies and increase biological control [58,59,60,61], but are much more difficult to integrate into vineyards due to their water requirements, competition with the grape vine, and interference with workers and machinery moving through the vineyard. Regardless, a small number of growers still experiment with various flowering summer cover crops (Figure 1), which include buckwheat (*Fagopyrum esculentum*), sweet alyssum (*Lobularia maritima*), purple tansy (*Phacelia tanacetifolia*), and clovers (*Trifolium* spp.). While the use of summer flowering cover crops is still relatively rare in California, primarily because of irrigation requirements, demand is sufficient for many seed companies to offer various “insectary blends” of flowering cover crop seeds. The effect of summer flowering cover crops on biological control in vineyards is unclear, and studies have not produced consistent outcomes [58,59,60,61,62,63,64]. In some cases, changes in pest densities in the presence of flowering cover crops was related to changes in vine vigor induced by competition from the cover crop [53].

Another popular approach to habitat provision in vineyards is the establishment of hedgerows. These are usually positioned at the periphery of vineyards, and typically consist of perennial grasses, flowers, shrubs, and trees. Water use is still a concern for many growers, so drought-tolerant and/or California-native plants are preferred. Some common hedgerow plants include coyote brush (*Bacharris pilularis*), sage (*Salvia* spp.), yarrow (*Achillea millefolium*), *Ceanothus* spp., willow (*Salix* spp.), toyon (*Heteromeles arbutifolia*), elderberry (*Sambucus* spp.), and perennial buckwheat (*Eriogonum* spp.) [48]. Hedgerows are typically linear in form, although in some instances they have been established as “flower islands” within the vineyard [65]. Since they do not interfere with production as much, hedgerows are amenable to a wider segment of growers, although the costs of installation and maintenance are high, and they require large areas of land. As such, hedgerow placement is typically based on convenience, being placed in areas where wine grapes cannot be grown, rather than systemically integrated into a production system. As with cover crops, the impact of hedgerows on vineyard pests has produced mixed results, and requires further study [66,67,68].

Vineyard polycultures are very rare, and are generally not intentionally arranged to promote biological control, but they do exist, and could potentially influence arthropod communities. Where present, the most common addition is a second perennial crop, such as olives, apples, or pears, since much of the same vineyard machinery and implements can be used on these crops if arranged and/or trellised properly. Similarly, polycultures such as this exist where one crop is in the process of being replaced by another (e.g., conversion of pear orchard to vineyard). This is followed by a much smaller number of growers who have developed areas of mixed vegetable production, typically for a restaurant associated with the winery, although some do market their produce off-farm. In all cases, the various crops are typically differentiated into separate monoculture blocks, although there are a few instances in which growers do plant crops between vine rows (e.g., wheat crop grown in the row middles). When this is done, the additional crop is typically arranged in the center of the row middle in order to allow tractor tires to pass unobstructed on either side.

### 2.5. Natural Enemy Augmentation

In the absence of adequate habitat to support natural enemies, various species of parasitoids and/or predators are available to growers for purchase from commercial insectaries [69]. Most common in wine grape production is the use of predatory mites (e.g., *Galendromus occidentalis*, *Phytoseiulus persimilis*, *Mesoseiulus longipes*), predatory thrips (*Aeolothrips* spp.), lacewings (*Chrysoperla* spp.), mealybug destroyer (*Cryptolaemus montrouzieri*), and the vine mealybug parasitoid *Anagyrus pseudococci*. These predators and parasitoids are typically deployed as target pest densities begin to rise. Growers may also make inoculative releases early in the season with the hope of establishing a resident natural enemy population in the vineyard, although this practice is rare. Generally, natural enemy augmentation in agriculture has been most effective under greenhouse settings where organisms are confined to a small area, while the release of natural enemies under field conditions, including vineyards, has produced far more variable results [70,71]. For example, the augmentative release of the parasitoid *Anagyrus pseudococci* has been shown to reduce vine mealybug populations [34], while lacewing releases were never able to reliably reduce leafhopper densities [72,73]. Such variable outcomes can likely be attributed to the complexity of predator–prey interactions, especially under field conditions [74,75,76,77]. At present, few vineyards claim to rely exclusively on natural enemy augmentation, and in many cases, this approach is typically a first attempt at pest control prior to the use of chemical controls, which in many cases are still needed.

There are also some more novel forms of pest control that involve arthropod augmentation. For instance, the introduction of non-economic phytophagous Willamette mites (*Eotetranychus willametei*) onto grape vines has been shown to induce a plant defense response that effectively reduces the populations of the economically important Pacific spider mite (*Tetranychus pacificus*) [78,79], although this is rarely, if ever, actually done in vineyards. There have also been studies to evaluate the use of herbivore-inducted plant volatiles to attract natural enemies into vineyards, which has so far produced mixed results [80,81,82,83].

### 2.6. Animal Integration

Vertebrate animals can serve various functions in crop production. For instance, they can be used as a basic source of traction to pull implements, their manure can improve soil fertility, and their feeding can contribute to the biological control of pests.

Most common in vineyards is the provision of nest sites for birds. Raptor perches and nest boxes can attract predatory birds such as kestrels (*Falco sparverius*) and barn owls (*Tyto alba*), which can help control rodent and bird populations that damage grape vines and feed on the fruit, respectively. Nest boxes can also attract insectivorous songbirds such as western bluebirds (*Sialia mexicana*), tree swallows (*Tachycineta bicolor*), and chipping sparrows (*Spizella passerina*), which can contribute to biological control of insect pests. However, their diets are likely to be somewhat indiscriminate, and so they may consume beneficial insects as well [84]. Similarly, some growers have installed bat boxes to promote bat activity, which has the potential to contribute to the biological control of insect pests [85].

**Figure 2:**
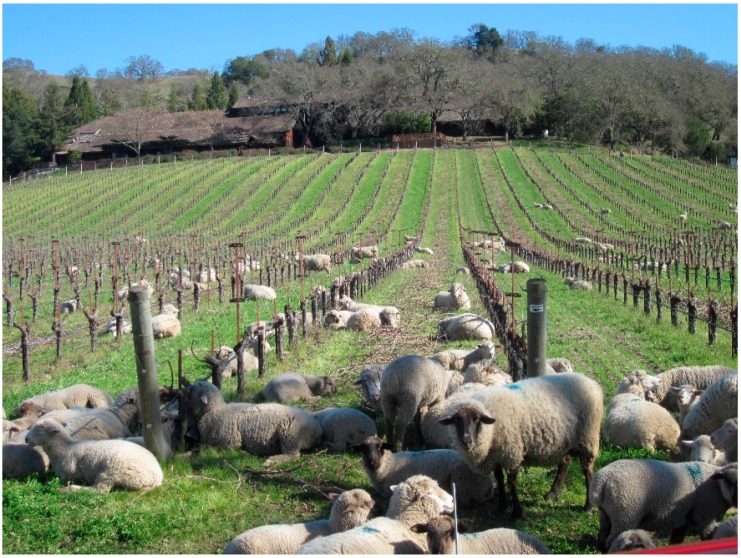
Sheep grazing on winter ground covers in a vineyard during the early spring.

Some vineyards bring in herds of sheep in the spring to graze down overwintering cover crops (Figure 2) [86]. As the sheep consume the cover crop, they deposit manure, which improves soil fertility. While sheep are not primarily used as a pest management strategy, they may inadvertently consume pests residing on the cover crop, such as leafhoppers, mites or thrips, although this has never been evaluated.

A very small number of growers are starting to experiment with the use of chickens for pest control as well. After mowing cover crops in the spring, large numbers of chickens are confined in the vineyard with the hope that they will feed on insects overwintering in the ground covers. As they hunt for insect prey, their scratching action may improve soil quality as well.

### 2.7. Biodynamic Preparations

Biodynamic agriculture has recently been growing in popularity as a more progressive alternative to the USDA-NOP. In addition to exclusively utilizing organic methods, biodynamic farms are also required to implement certain farming practices that may have implications for pest control, such as the production of multiple crop species, integration of animals into the production process, and maintenance of significant reserves of natural habitat in and around the farm [87,88,89]. Biodynamic farms also apply various “preparations” that are thought to improve crop productivity, increase soil microbial activity, and reduce pest and disease incidence [88,89,90]. Each preparation is a water extraction of specific plant and/or animal material; for instance, preparation 500 is a compost tea derived from cow manure, preparation 503 is derived from chamomile blossoms, and so on. None of the preparations specifically target insect pests, but rather seek to improve soil quality and, through this, increase plant health and resistance to pests and diseases. As such, preparations are essentially applied preemptively to ward off pest and disease problems, rather than in reaction to a localized pest or disease outbreak. The use of biodynamic farming practices has been shown to improve vine balance [91] and affect wine grape quality [92,93], but little is known about the impacts of these practices on pest and disease pressure in vineyards [90].

## 3. Conclusions and Future Directions

Arguably the largest reduction in vineyard pesticide use (and non-target impacts) can be attributed to the widespread adoption of IPM practices, paired with the development of more novel pesticide chemistries and improved application technology. That said, many IPM vineyard systems still regularly rely on multiple applications of chemical controls for pest and disease management. As such, there are still additional gains to be made, and EBPM could hold the key. At present, the most conspicuous EBPM practices involve pheromone mating disruption, the augmentative releases of natural enemies, and on-farm habitat diversification to enhance the biological control of vineyard pests (Table 1). The use of such EBPM practices is most common in vineyards where chemical control options are limited (i.e., certified organic and/or biodynamic vineyards), but there is also a large contingent of “conventional” growers who do not hold any ecological certification that make use of EBPM as well.

Few growers would claim to exclusively rely on EBPM, although some do. Such partial adoption is due to the fact that many EBPM practices tend to be more costly than chemical control options and do not produce as consistent results. Thus, the degree to which a wine grape grower relies on EBPM practices is heavily determined by economic and ecological context. California wine grape production is distributed across multiple regions throughout the state, and the fruit is produced for a variety of markets (e.g., bulk wine, fine wine, etc.). Regional differences in climate and landscape diversity can influence pest pressure, while land rents and crop value determine grower tolerance for pests and treatment thresholds [14,94,95]. In this way, a grower’s ability to adopt EBPM strategies over chemical controls can be greatly influenced by the location of their vineyard and/or their target market. At the same time, grower testimonials and anecdotal evidence of the success of their EBPM practices may also be obscured in regions with low overall pest pressure and/or high profit margins, the latter of which allows growers to tolerate higher levels of pests and diseases as well as crop damage. For instance, growers in the North Coast region produce high-value grapes for fine wine in a landscape with large patches of natural habitat surrounding the vineyard due to restrictions on hillside vineyard development. Agricultural regions with high levels of landscape diversity tend to have lower overall pest pressure [96,97], and it may be that growers who utilize EBPM in such regions incorrectly attribute lower pest densities to their on-farm efforts, rather than to the influence of landscape diversity [59].

This is not to say that EBPM practices do not or cannot reduce/replace pesticide use in California wine grape production. Rather, the limited development and adoption of reliable EBPM practices can be traced to a lack of scientific research, which is further reinforced by economic impediments to adoption. Some of the economic barriers are alleviated through the Environmental Quality Incentives Program (EQIP), which was created as part of the 1996 farm bill. The EQIP program provides cost-share to growers interested in the implementation of on-farm practices and improvements that reduce the negative impacts of agriculture and promote biodiversity conservation, such as, for instance, the installation of perennial hedgerows and use of flowering cover crops. A key limitation of this program is that it does not support follow-up monitoring or evaluation of impacts, so the degree to which these subsidized diversification practices are actually reducing pesticide use in vineyards remains unclear. As a result, their efficacy remains an outstanding question for many EBPM practices. A lack of research funding has allowed for the persistence of knowledge gaps regarding the design and implementation of EBPM strategies that can produce reliable results [98]. Further complications arise from the fact that EBPM practices are inherently specific to the pest, crop, and even region [99,100,101], which places a strain on the broader adoption and scalability of research findings.

By subsidizing the implementation of EBPM practices that have not yet been fully elaborated, programs like EQIP are putting the cart before the horse. A priority for policy-makers should be the creation of additional funds for research on EBPM. The development of more reliable EBPM practices would likely promote adoption, which could lead to reduced pesticide use in California wine grape production. Research priorities should focus on a variety of areas, such as the development and use of botanical pesticides, additional modes of pest mating disruption, alternative ant bait delivery techniques, natural enemy augmentation strategies, habitat management for conservation biological control, and studies on the efficacy of biodynamic practices and impacts of animal integration on biological control. Wine grape growers across the board are very interested in EBPM as an alternative to chemical controls, but without a set of reliable and affordable practices, many growers are hesitant to adopt them. Without further research, such reliable and affordable practices will never exist.

## Figures and Tables

**Table 1:** Estimated adoption rate (as % of total acres) of integrated pest management (IPM)/ecologically-based pest management (EBPM) strategies in California wine grape vineyards.

Practice	Details	Estimated Adoption (% Total Acres)
Spray timing and calibration	Travel speed, nozzle position, boom orientation, wind conditions, etc.	85–95%
Monitoring	Weekly scouting, pest identification, use of economic thresholds, etc.	70–80%
Least toxic product selection	Guidelines from UC IPM, IOBC, etc.	85–95%
Pheromone mating disruption	Primarily vine mealybug (*Planococcus ficus*)	10–15% (when needed)
Ant bait stations	Mealybug control (*Pseudococcus* spp., *Planococcus ficus*)	<5%
Natural enemy augmentation	Predatory mites (*Galendromus occidentalis*, *Neoseiulus californicus*), lacewings (*Chrysoperla* spp.), beetles (*Cryptolaemus montrouzieri*) etc.	<5%
Habitat management	Overwintering cover crops	75–85%
	Perennial grass cover to reduce vigor	60–70% (when needed)
	Mustards for nematode control	10–20% (when needed)
	Hedgerow	<5%
	Summer flowering cover crop	<2%
Animal integration	Bird/bat boxes, raptor perches	5–10%
	Sheep, chicken or other grazing	<5%
Biodynamic preparations		<2%
